# Local measles vaccination gaps in Germany and the role of vaccination providers

**DOI:** 10.1186/s12889-017-4663-3

**Published:** 2017-08-14

**Authors:** Linda Eichner, Stephanie Wjst, Stefan O. Brockmann, Kerstin Wolfers, Martin Eichner

**Affiliations:** 1 Institute for Clinical Epidemiology and Applied Biometrics, University Tübingen, Silcherstraße 5, 72076 Tübingen, Germany; 2 Public Health Office Reutlingen (Landratsamt Reutlingen), Gesundheitsamt, St.-Wolfgang-Straße 13, 72764 Reutlingen, Germany

**Keywords:** Measles, Vaccination, Vaccination gaps, Immunization rate, Kindergarten

## Abstract

**Background:**

Measles elimination in Europe is an urgent public health goal, yet despite the efforts of its member states, vaccination gaps and outbreaks occur. This study explores local vaccination heterogeneity in kindergartens and municipalities of a German county.

**Methods:**

Data on children from mandatory school enrolment examinations in 2014/15 in Reutlingen county were used. Children with unknown vaccination status were either removed from the analysis (best case) or assumed to be unvaccinated (worst case). Vaccination data were translated into expected outbreak probabilities. Physicians and kindergartens with statistically outstanding numbers of under-vaccinated children were identified.

**Results:**

A total of 170 (7.1%) of 2388 children did not provide a vaccination certificate; 88.3% (worst case) or 95.1% (best case) were vaccinated at least once against measles. Based on the worst case vaccination coverage, <10% of municipalities and <20% of kindergartens were sufficiently vaccinated to be protected against outbreaks. Excluding children without a vaccination certificate (best case) leads to over-optimistic views: the overall outbreak probability in case of a measles introduction lies between 39.5% (best case) and 73.0% (worst case). Four paediatricians were identified who accounted for 41 of 109 unvaccinated children and for 47 of 138 incomplete vaccinations; GPs showed significantly higher rates of missing vaccination certificates and unvaccinated or under-vaccinated children than paediatricians.

**Conclusions:**

Missing vaccination certificates pose a severe problem regarding the interpretability of vaccination data. Although the coverage for at least one measles vaccination is higher in the studied county than in most South German counties and higher than the European average, many severe and potentially dangerous vaccination gaps occur locally. If other federal German states and EU countries show similar vaccination variability, measles elimination may not succeed in Europe.

**Electronic supplementary material:**

The online version of this article (doi:10.1186/s12889-017-4663-3) contains supplementary material, which is available to authorized users.

## Background

Measles is highly contagious and claims many lives every year, particularly among young children. Annually, approximately 135 million cases of measles and 6 million measles-related deaths occurred worldwide before vaccination was introduced in 1963 [1]. Global measles control has been very successful: in countries with routine measles immunization, mass vaccination campaigns, and appropriate case management, measles deaths have dropped dramatically between 2000 and 2008, from 733,000 to 164,000 [1]. According to WHO surveillance data, 189,844 measles cases were reported worldwide in 2016, and annual measles deaths have declined by 79% from the year 2000 to 134,200 deaths in 2015 [2, 3]. In the European Economic Area (EU/EEA), there were 1818 measles cases from 1 July 2015 until 30 June 2016, 309 of which were reported from Germany [4]. In Germany alone, 442 to 1764 cases occurred annually from 2011 to 2015 (5.5–21.9 per million), many of them in Berlin and in the southern states of Baden-Württemberg (BW) and Bavaria [5]. The most recent epidemic in Berlin lasted from October 2014 to August 2015 and led to 1359 cases [6]. In 2015, 111 measles cases were reported in BW [7]. The revised European Vaccine Action Plan, EVAP 2015, aims for measles elimination [8]. Key challenges include vaccination hesitancy, complacency, and under-served populations [8]. Germany ensured its commitment by establishing a national action plan for 2015–20 as well as a national verification commission for the elimination of measles and rubella [9].

In Germany, measles is a notifiable disease; vaccinations are not mandatory and can only be administered by physicians. Parents can choose whether their children are registered with paediatricians or general practitioners. Due to a shortage of physicians in rural areas, some parents may opt for their children to be registered with general practitioners rather than paediatricians. According to the German Standing Committee on Vaccination (Ständige Impfkommission, STIKO), children should receive their first measles vaccination at the age of 11–14 months. A second vaccination is to be administered between the ages of 15 and 23 months [10].

Children can be enrolled in schools without proof of vaccination or immunity, but the local public health offices have to survey the vaccination certificates of all 4- to 5-year-old children during the mandatory school enrolment examinations (Einschulungsuntersuchungen, ESU) [11]. The German national vaccination coverage during the school enrolment examination in 2014 was reported as 96.7% for one vaccination and 92.4% for two vaccinations. In comparison, BW had 95.0% coverage for one and 89.1% for two vaccinations [12]. Recent legal amendments aim to improve the vaccination coverage of children: before they can be enrolled in kindergarten, their parents must now consult a physician who has to certify that they were informed about vaccinations. Their proof of consultation is not documented in the vaccination certificate but on a separate document, which is to be presented to the kindergarten or school upon enrolment, whether the institution is public or private [11] . There is no standardized vaccination surveillance system in Germany. Thus, the assessment of the vaccination and immunity status of the population has to use random samples or cross sectional surveys. The only routinely collected data on vaccination is obtained by mandatory school enrolment examinations and by the newly established national “KV-Impfsurveillance” project, which assesses the vaccination records of health insurance companies [13].

The aim of this study is to assess the local variability of measles vaccination coverage in the German county of Reutlingen (BW) at a small community level based on the data collected from 4- to 5-year-olds during the ESU in the 2014 and 2015 school year. Under-vaccinated pockets will be identified and can then be targeted through tailored vaccination action plans.

## Methods

This study is based on measles vaccination data of 4- to 5-year-old children from 2014 and 2015 in Reutlingen county that were routinely collected by us. Reutlingen county is located in the south German state of BW and has a population of 278,031 inhabitants, who live in 26 municipalities with 199 kindergartens. During ESU, the local public health office routinely records the children’s physicians and vaccination status by examining the children’s vaccination certificates presented during the examination at the local public health office, as demanded by the German National Protection Against Infection Act (Infektionsschutzgesetz, §34 IfSG) [11], by the State Public Health Law (Gesundheitsdienstgesetz, §8 ÖGDG) [14], and by the Ministry of Health’s Administrative Regulations [15]. Therefore, the data that were collected and anonymized by the authors could be used freely for this work and ethical approval was not required. The dataset includes the age, gender, measles vaccination status, physician, kindergarten, and residence municipality of each child.

Since some of the children did not present a vaccination certificate, the data were analysed twice: in our “worst case” analyses, it was assumed that children without vaccination certificates were not vaccinated, while in our “best case” analyses, such children were excluded (implicitly, this assumes that they had the same average vaccination status as the others). The vaccination coverage of children with at least one measles vaccination and with both measles vaccinations were calculated for each municipality and kindergarten in the county of Reutlingen. Kindergartens and municipalities with data on <10 children were omitted from some of the analyses.

To examine the presence of a vaccination certificate or the vaccination status, children were classified in different groups: [1] children who were registered with GPs and [2] children who were registered with paediatricians. Fisher’s exact test was used to compare these groups. A third group of children, whose parents were not able to supply the name of a physician, was considered in the multivariate analysis, but not in the group comparisons.

In a measles outbreak in a German school, 98.1% of vaccinees were protected against infection after one vaccination (vaccine efficacy VE_1_) and 99.4% after two (VE_2_); for this outbreak, the basic reproduction number (R_0_) of measles (i.e., the expected number of secondary cases in a completely susceptible population caused by a single measles case) in a school setting was estimated to be 30.1 [16]. Multiplying the vaccine efficacy (VE) by the vaccination coverage of a community results in the average immunity level (x). In a population where a proportion x is immune, the expected number of secondary infections reduces to the “effective reproduction number,” R_e_ = R_0_(1-x), which can be used to calculate the probability P_e_ = 1/R_e_ that an epidemic occurs if measles is introduced into the population [17]. Although this probability is valid only for large populations, it can be used as an indicator of how well a group of individuals is protected against the continued spread of measles. To calculate the percentage that must be vaccinated to prevent epidemics, the immunity level x must reduce R_e_ to a value less than 1, i.e., x > 1–1/R_0_. Using R_0_ = 30.1, x must exceed 96.7%. To obtain this level of immunity by a single vaccination (VE_1_ = 98.1%), 98.6% of the children must be vaccinated; for two vaccinations (VE_2_ = 99.4%), 97.3% must be vaccinated.

To identify physicians and kindergartens with excessively high percentages of unvaccinated children, we performed univariate analyses as follows: for each physician (and for each kindergarten), [1] the number n of all children with vaccination certificates who were registered with the physician (or who attended the kindergarten) was determined, [2] the number k of these children who were not vaccinated was determined, [3] the probability P that children with vaccination certificates were not vaccinated was calculated for all other children who were not registered with the physician (or who did not attend the kindergarten). [4] The probability that at least k out of n children randomly failed to be vaccinated, even though they had exactly the same probability P as the others, could then be calculated with the binomial distribution. [5] Physicians and kindergartens whose resultant probabilities that their children were unvaccinated was rather unlikely (i.e., below 1/25) were identified and were later included in the bivariate analyses. In these bivariate analyses, we examined the joint influence of physicians and kindergartens. We first formulated the null hypothesis that each child with a vaccination certificate (irrespective of his or her physician or kindergarten) had exactly the same probability of being vaccinated. To challenge this null hypothesis, we assumed that the child’s probability of being unvaccinated furthermore depended on his or her physician and kindergarten (for a full description of the model, see Online Additional file 1). Parameters were estimated by maximum likelihood. Calculating 95% confidence intervals for each additive term of the physicians and kindergartens allowed an assessment of whether these influences were statistically significant. The same series of univariate and bivariate analyses was then repeated with children who were vaccinated at least once (n) and with fully vaccinated children (k). The statistical package JMP was used for data evaluation [18].

## Results

**Fig. 1:**
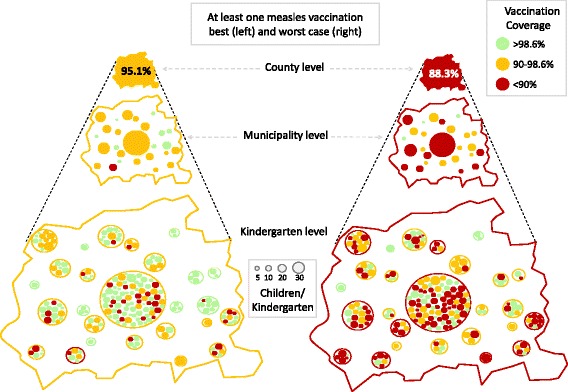
Vaccination coverage with at least one measles vaccination, County Reutlingen (worst case and best case). Illustration of the vaccination coverage (for at least one vaccination) on different community levels. Data were collected in 2014/15 in County Reutlingen (Baden-Württemberg, South Germany) during school enrolment (ESU). The figures display the best-case scenario, where all children without vaccination certificates were omitted from analysis (left), and the worst-case scenario (right), where these children were regarded as unvaccinated. The local vaccination coverage was colour-coded (see inlet). The areas of the kindergartens are proportional to the number of children for whom data were available (see inlet). To ensure the anonymity of kindergartens, the dots do not represent real geographic locations

**Fig. 2:**
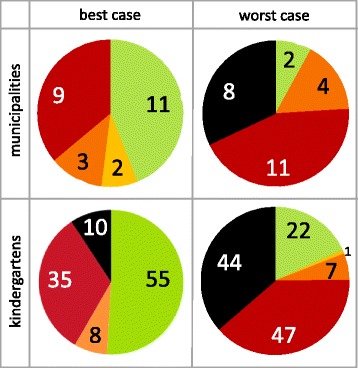
Measles epidemic probabilities in municipalities and kindergartens. Number of municipalities (*top*) and number of kindergartens (*bottom*) in which an introduced measles infection is expected to cause an epidemic with a probability of 0% (*green*), >0–25% (*yellow*), >25–50% (*orange*), >50–75% (*red*), or >75% (*black*). Left: best case; right: worst case; municipalities and kindergartens with <10 children were excluded

A total of 170 (7.1%) of 2388 children of the 2014/15 ESU cohort did not present a vaccination certificate. In terms of physicians, 2099 of the children were registered with 56 paediatricians, while the remaining 289 were registered with 45 general practitioners (GPs). Vaccination coverage values for at least one measles vaccination in County Reutlingen, its municipalities and kindergartens are visualized in Fig. 1. Some of the kindergartens and municipalities are highly under-vaccinated, making them vulnerable to measles introduction (Figs. 1 and 2 and Table 1).

**Table 1:** Vaccination coverage for at least one vaccination among 4- to 5-year-olds in Reutlingen county

	Best-case scenario		Worst-case scenario	
		Range of coverage		Range of coverage
In kindergartens	*n* = 108	From 52.5% to 99.4%	*n* = 121	From 42.5% to 89.5%
In municipalities	*n* = 24	From 66.1% to 99.4%	*n* = 25	From 55.1% to 94.7%
In the county		95.1%		88.3%

In the worst-case analyses, children without vaccination certificates were regarded as unvaccinated, and, thus, all children could be used. In the best-case analyses, such children were excluded from the analysis, leading to different estimates of vaccination coverage. In the sub-analyses, only municipalities (and respectively kindergartens) with at least 10 children (worst case estimates) or with at least 10 children who presented vaccination certificates (best case estimates) were used

Significantly higher percentages of children who were registered with a GP had no vaccination certificate (11.8 vs. 3.8%), were unvaccinated (9.4 vs. 4.7%), or were vaccinated only once (9.6 vs. 6.1%) compared to children who were registered with a paediatrician (*p* < 0.001). In our first univariate analyses, two kindergartens (out of 199) and four paediatricians (out of 56) with exceptionally high fractions of unvaccinated children were identified (see Online Additional file 1 for details). As some children who attended one of these two kindergartens were also registered with one of the four paediatricians, we added a bivariate analysis, which allowed for competing risks. This reduced the number of identified kindergartens to one, whereas all four paediatricians significantly increased the children’s baseline probability of being unvaccinated. In a second univariate analysis, eight kindergartens and three paediatricians with exceptionally high fractions of incompletely vaccinated children were identified. The subsequent bivariate analysis reduced the number of kindergartens to five, whereas all three paediatricians significantly increased the children’s baseline probability of being incompletely vaccinated. Interestingly, these three paediatricians are a subgroup of the four paediatricians who were identified in the first analyses (concerning children who were unvaccinated). The fourth paediatrician may only have dropped out of the second analysis (concerning complete vaccination) because too few children with at least one vaccination were left. Altogether, 37.6% (41/109) of all unvaccinated children and 34.1% (47/138) of all incompletely vaccinated children (best-case scenario) were registered with these four paediatricians.

## Discussion

**Fig. 3:**
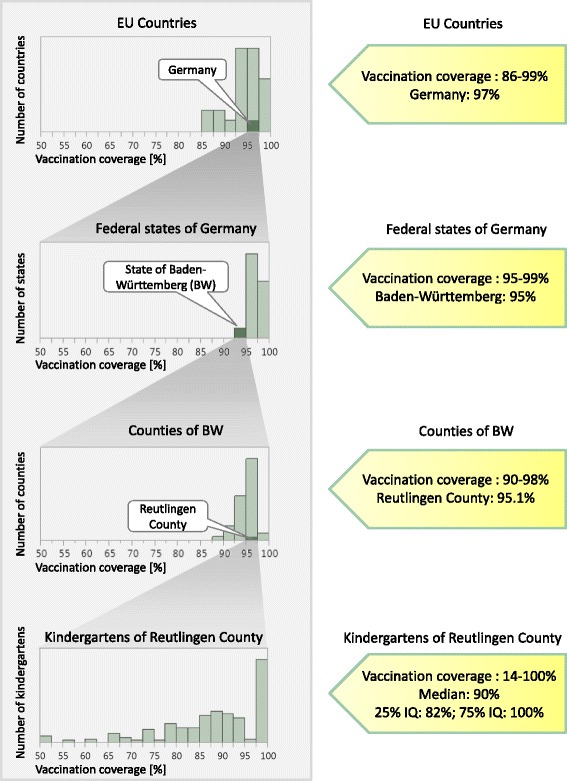
Frequency of communities grouped by measles vaccination coverage (best-case scenario; at least one vaccination; 2013–15). Level 1: comparison of school enrolment (ESU) vaccination coverage of Germany with other EU countries (data from WHO 2014 [19]; data from Austria and Czech Republic were missing; no data were found for Finland and Poland for 2014, so data from WHO 2013 were used instead for these two countries). Level 2: comparison of Baden-Württemberg (BW) to the other federal states of Germany [12]. Level 3: comparison of County Reutlingen to the other counties of BW [31]. Level 4: vaccination coverage of the kindergartens of County Reutlingen, ranging from 14 to 100%

The average population-weighted vaccination coverage of the EU/EEA for at least one measles vaccination is 93.7% [19]; to calculate this average, the population sizes of the countries were taken into account. With 96.7% coverage, Germany is among the better vaccinated countries: only five countries have a higher coverage (Fig. 3). Although the state of Baden-Württemberg (vaccination coverage 94.8%) is at the lower end of the vaccination scale in Germany, its vaccination coverage is still higher than the EU/EEA average. It can be seen that – although Reutlingen county has an overall coverage of 95.1% – vaccination coverage at a kindergarten level is very heterogeneous (14–100%; Fig. 3). If we assume that other federal German states or EU countries show similar vaccination variability at community and kindergarten levels (cf. Fig. 1), measles elimination may not succeed in Europe. This variability would also explain why apparently well-vaccinated populations still experience measles outbreaks. In 2006, there were 94 measles cases in Canada, despite 95% coverage for one measles vaccination [20]. In the same year, Catalonia, Spain, which has 97% national vaccination coverage with one dose, reported an outbreak of 381 cases [19, 21]. A more recent study from the Netherlands reported 1226 measles cases in 2013. The national vaccination coverage was 95% for one dose [22]. This shows that high national or regional vaccination coverage cannot guarantee the prevention of outbreaks. All of these outbreaks were linked to communities that had low vaccination coverage. Many of the affected people were not vaccinated. A modelling study on the measles vaccination coverage of children at school enrolment in San Diego County, USA, found that heterogenic vaccination coverage in a school setting increased R_e_ by 70%, which increased the probability of outbreaks. Reaching under-vaccinated groups in schools and improving their vaccination uptake would greatly decrease the chance of outbreaks [23]. These studies demonstrated the dangers of under-vaccinated pockets, even in countries with generally high vaccination coverage (>95%), and showed the importance of the timeliness of vaccinations and catch-up vaccinations.

To increase infant and child vaccination coverage, the WHO advises tailoring immunization programmes (TIP) [24]. In our search for vaccination gaps, we have identified four paediatricians (out of 56) and six out of 199 kindergartens with extremely low vaccination rates. The fact that many of the under-vaccinated children belonged to few paediatricians may partly be due to geographical and/or social clustering effects. As parents are free to choose paediatricians of their own liking, families with reservations against vaccination may cluster with some paediatricians and avoid others. GPs provide medical treatments to all age groups and are less specialized for treating children. Therefore, they may be less informed on current vaccination schedules for children and may have a shortage of the vaccines needed for children. Some GPs may even neglect asking about a child’s vaccination status or offering a vaccination under the assumption that the child’s paediatrician has already done so. These factors could explain why children seeing GPs have a lower vaccination status than those seeing paediatricians.

The outcomes of this work have stimulated discussions on the current situation and on targeted solutions. Although physicians themselves may not always be the cause of under-vaccination, they could still be pivotal points of intervention campaigns. All paediatricians of County Reutlingen have been informed of their vaccination results and their ranking among their peers. The vaccination performance of physicians in County Reutlingen and the consequences thereof, in terms of vaccination gaps at a small community level, were also presented to and discussed with the Medical Association of BW (Landesärztekammer) and the mayors of the communities, so that this issue can be approached both on a large and small community level. Vaccination coverage could be largely increased by improving the vaccination uptake of the children who were registered with the four identified paediatricians. Even if the vaccination status of only these children reached the level of the others, 29.6% of all unvaccinated children and 36.9% of all incompletely vaccinated ones would be prevented.

Children registered with GPs generally had lower vaccination rates than those registered with paediatricians. Unlike paediatricians, GPs could not be analysed individually because of the small number of children per GP (most of them observed less than 5 children). The issue of the low vaccination coverage of children who were registered with GPs should also be discussed in the German GP Associations. The German Umbrella Organization of the Paediatric Societies (DAKJ) also demanded that the German National Medical Chamber should pursue legal actions against physicians who fail to comply with the German National Vaccination Recommendations. They further demand that children may only enrol in private or public kindergartens and schools if they have received all vaccinations (unless a contraindication exists) that are recommended in the German vaccination calendar by STIKO [25]. Given the compulsory school attendance in Germany, this would imply obligatory vaccination, as was recently demanded by the president of the German National Medical Chamber [26]; yet, so far, compulsory vaccination has been declined in Germany.

A major problem that became apparent during this study was the huge gap between the best- and worst-case scenarios, which was caused by participants without vaccination certificates. It would be helpful if the existing legal obligation of providing a vaccination certificate at ESU was actually enforced. At the moment, it is common practice in Germany to calculate the vaccination coverage only from data on children who present a vaccination certificate [27, 28], which may easily lead to over-optimistic views. A primary necessary prerequisite for targeted strategies is a trustworthy picture of the population’s vaccination status, which demands the establishment of an immunization registry. Currently, the measles vaccination coverage of Germany seems to be exactly as high as in Sweden, yet this result may be misleading: the Swedish dataset is registry based, whereas the German data are based on a best-case scenario. The establishment of a vaccination register, per se, would not improve vaccination coverage, but it would help shed light on the vaccination status of the population and identify under-vaccinated pockets and sources of low coverage.

### Limitations

One limitation of this study is that we could only use data for 4- to 5-year-old children in the 2014/15 cohort in County Reutlingen; but, when looking at data from the previous years, the coverage of children who were vaccinated at least once varied only marginally (2012/13: 96.6%; 2013/14: 96.7%). Over the last sixteen years, vaccination coverage has gradually increased, and it has reached a plateau in Reutlingen country. This also implies that older children and juveniles may have an even lower vaccination coverage than the children in this study. Another limiting factor is that only one German county was analysed in this study. The mean vaccination coverage of Reutlingen (95.1%) exceeds the mean of BW (94.8%), yet it lies below the mean of Germany (96.7%; Fig. 3). The vaccination status in other German counties may be quite different, but the same degree of heterogeneity must be expected there as well. When translating vaccination coverage into outbreak probabilities, we used R_0_ = 30.1, which is higher than other reported values [29, 30]. This can be explained by the fact that it was derived from a school outbreak: R_0_ commonly describes the potential spread of an infection in a whole population comprised of all ages, yet children and juveniles have much more contact (particularly among themselves) than adults. Accordingly, R_0_ values that are restricted to school children must be larger than population-based values, and infections spread faster in schools and kindergartens than in the population. Using lower values of R_0_, the calculated outbreak probabilities should decrease, yet this issue is at least partly compensated, as the vaccine efficacy that is used in this study has been estimated together with the value of R_0_ from the same measles outbreak in a school setting [16]. In Fig. 1, kindergartens with few children frequently have either very high (>98.6%) or very low vaccination coverage (<90%). This result is simply due to their sizes, and it can lead to an evaluation bias (it must not be interpreted as the result of different vaccination behaviours of small groups). We have tried to avoid this bias by excluding kindergartens and municipalities from the analyses when we looked at vaccination coverage within these communities.

## Conclusions

Missing vaccination certificates pose a severe problem regarding the interpretability of vaccination data. Although the vaccination coverage of the studied county is better than most South German counties and exceeds that of the European population, many severe and potentially dangerous vaccination gaps occur locally. Assuming that other German states and EU countries show similar variability, measles elimination may not succeed in Europe. Such gaps are best targeted by tailored immunization programmes that involve communities, kindergartens and physicians. A necessary prerequisite for targeted strategies is a trustworthy picture of the population’s vaccination status, which demands the establishment of an immunization registry.

## Additional file

Additional file 1: The file contains detailed information on the univariate and bivariate analyses of kindergartens and physicians. (DOCX 35 kb)

## Abbreviations

BW: Baden-Württemberg (a state in South Germany)
DAKJ: The German umbrella organization of paediatric societies
ESU: Mandatory school enrolment examinations
EU: European Union
EVAP: European Vaccination Action Plan
GP: General practitioners
IfSG: German National Protection Against Infection Act
JMP: Name of statistics software
P_e_: Probability of an outbreak
R_0_: Basic reproduction number
R_e_: Effective reproduction number
STIKO: German National Vaccination Recommendations
TIP: Tailored Immunization Program
VE_1_: Vaccine efficiency for one dose of the measles vaccine
VE_2_: Vaccine efficiency for two doses of the measles vaccine
WHO: World Health Organization
