# Supplementary material for: Local measles vaccination gaps in Germany and the role of vaccination providers
Source: BMC Public Health. 2017 Aug 14;17:656. doi: 10.1186/s12889-017-4663-3 (PMC5557556; doi:10.1186/s12889-017-4663-3)
Supplement: Additional file 1: — The file contains detailed information on the univariate and bivariate analyses of kindergartens and physicians. (DOCX 35 kb) [file 12889_2017_4663_MOESM1_ESM.docx]

**Appendix**

**Univariate analysis of kindergartens and physicians**

Our null hypothesis assumes that the vaccinated percentage of children was the same in every kindergarten (likewise for every physician). Accordingly, the probability of observing k unvaccinated children in a kindergarten where n children provided a vaccination pass is given by the binomial distribution:$\left(\begin{matrix} n \\ k \end{matrix}\right) p^{k} \left(1-p\right) ^{n-k}$ whereby p is the fraction of vaccinated children in all kindergartens combined (see above). To determine whether the observed number of unvaccinated children was particularly unlikely under this hypothesis, the expected fraction of kindergartens with n children who had better vaccination coverage (i.e. less than k unvaccinated children) was calculated; this was given by $\sum_{i=0}^{k-1} \left(\begin{matrix} n \\ i \end{matrix}\right) p^{i} \left(1-p\right) ^{n-i}$. Assuming that the result was 99%, the probability of having such bad results could be expressed as 1-0·99 = 1/100. Pediatricians and kindergartens were declared “conspicuous” if this probability was below 1/25. The same analysis was done for kindergartens and for physicians, whereby the event of interest was either “child presented a vaccination pass”, or “child was vaccinated at least once” or “child was fully vaccinated”.

**Bivariate analysis of kindergartens and physicians**

In order not to estimate too many parameters, only kindergartens and physicians which were identified in the previous section were included individually in the following bivariate analyses, whereas all other non-conspicuous kindergartens were grouped as “other KiGa” and all non-conspicuous pediatricians were grouped as “other Ped”. Further categories were “no KiGa”, “GP” and “no physician”. Table A1 gives an example of the framework of the model which was used for parameter estimation.

Table A1 Example for a bivariate public health forensics model.

| Model | Ped1 | Ped2 | other Ped | GP | no physician |
| --- | --- | --- | --- | --- | --- |
| KiGa1 | b+k_1_+p_1_ | b+k_1_+p_2_ | b+k_1_ | b+k_1_+g | b+k_1_+p_0_ |
| KiGa2 | b+k_2_+p_1_ | b+k_2_+p_2_ | b+k_2_+p_2_ | b+k_2_+g | b+k_2_+p_0_ |
| other KiGa | b+p_1_ | b+p_2_ | **b** | b+g | b+p_0_ |
| no KiGa | b+k_0_+p_1_ | b+k_0_+p_2_ | b+k_0_ | b+k_0_+g | b+k_0_+p_0_ |

In this example, two kindergartens (KiGa1 and KiGa2) and two pediatricians (Ped1 and Ped2) were identified in the preceding univariate analysis to have unduly contributed to missing vaccinations. Terms in the table show the com­bination of parameters which needed to be estimated by maximum likelihood (see text).

Children in the combined category “other Ped” (all non-conspicuous pediatricians combined) and “other KiGa” (all non-conspicuous kindergartens combined) were used as reference category for which the baseline probability “b” was estimated. In all other fields of Table A1, additional probabilities were added, depending on the combination of physician and kindergarten. Parameters k_0_ and p_0_ allow calculating different probabilities for children without kindergarten or without physician. Parameter g allows that GPs have a different probability than pediatricians. All parameters except for b were can obtain negative values (indicating protective effects). For each field of Table A1, the observed number n of children and the number k of children with unwanted event (e.g. no vaccination) were given from the data. The likelihood of each field of Table A1 (e.g. probability p=b+k_1_+p_1_) was given by the binomial distribution as $\left(\begin{matrix} n \\ k \end{matrix}\right) p^{k} \left(1-p\right) ^{n-k}$. The total likelihood of all observations was given by multiplying the likelihoods obtained for all fields. All probabilities were jointly estimated by maximum likelihood, using the statistics software JMP. Furthermore, 95% confidence intervals were calculated for each parameter. If e.g. the confidence interval of p_1_ (additional probability for Ped1) contained negative values, “Ped1” may have had a protective rather than aggravating effect, and was removed from the list of conspicuous pediatricians (likewise for kindergartens). The event of interest in these analyses was either “child presented a vaccination pass”, or “child was vaccinated at least once” or “child was fully vaccinated”.
